# Optimal Energy Efficiency Fairness of Nodes in Wireless Powered Communication Networks

**DOI:** 10.3390/s17092125

**Published:** 2017-09-15

**Authors:** Jing Zhang, Qingjie Zhou, Derrick Wing Kwan Ng, Minho Jo

**Affiliations:** 1 School of Electronic Information and Communications, Huazhong University of Science and Technology, Wuhan 430074, China; zhangjing@hust.edu.cn (J.Z.); m201771970@hust.edu.cn (Q.Z.); 2 School of Electrical Engineering and Telecommunications, University of New South Wales, Sydney 2052, Australia; wingn@ece.ubc.ca; 3 Department of Computer Convergence Software, Korea University, Sejong Metropolitan 30019, Korea

**Keywords:** energy efficiency, fairness, WPCN, energy harvest, wireless power transfer

## Abstract

In wireless powered communication networks (WPCNs), it is essential to research energy efficiency fairness in order to evaluate the balance of nodes for receiving information and harvesting energy. In this paper, we propose an efficient iterative algorithm for optimal energy efficiency proportional fairness in WPCN. The main idea is to use stochastic geometry to derive the mean proportionally fairness utility function with respect to user association probability and receive threshold. Subsequently, we prove that the relaxed proportionally fairness utility function is a concave function for user association probability and receive threshold, respectively. At the same time, a sub-optimal algorithm by exploiting alternating optimization approach is proposed. Through numerical simulations, we demonstrate that our sub-optimal algorithm can obtain a result close to optimal energy efficiency proportional fairness with significant reduction of computational complexity.

## 1. Introduction

With the wide use of smartphones, tablets, and machine-to-machine (M2M) devices for various applications and services, the amount of mobile data traffic has grown dramatically in recent years [1]. The deployment of low-power small base stations (BSs) in hotspot areas is a potential solution to cope with the increase in traffic and devices [2]. In particular, with the density of low-power small BSs, heterogeneous cellular networks (HetNets) could enhance area spectral efficiency, increase the capacity of communication, and reduce transmission delay [3]. Therefore, a high dense heterogeneous network is one of the preeminent technologies in the racetrack towards fulfilling the requirements of next generation mobile networks [4].

In practice, low-power small BSs have advantages on both devices and networks in terms of energy efficiency. On the one hand, low-power small BSs serving wireless devices with short communication distances results in a lower power consumption. On the other hand, the BSs of a macrocell can reduce power consumption by offloading part of the traffic to small BSs. As a result, various energy-efficient designs have been proposed [5,6,7,8,9] to exploit the potential performance gains brought by deployment of low-power small BSs. A novel approach for joint power control and user scheduling has been proposed in [5] for optimizing energy efficiency (EE) while ensuring user QoS in ultra-dense small cell networks (UDNs). An algorithm is proposed in [6] to enable small cells activation/deactivation adaptively with respect to the dynamically fluctuating traffic loads to fullfil the data rate requirement. The user association problem is investigated in [7] for maximizing the energy efficiency of a network considering the capacity and energy consumption and a low-complexity algorithm achieving near-optimal performance is proposed. A non-convex optimization problem is formulated for power control of small BSs to maximize their EE in [8] and a distributed power control scheme is proposed to achieve the Nash equilibrium with the minimum information exchange. The energy efficiency of the uplink transmission in HetNets, where the user equipments (UEs) apply a flexible power control scheme subject to maximum transmit power constraint, is investigated in [9]. Meanwhile, there has also been growing interest in studying various techniques for the nodes to improve the energy efficiency of the network [10,11,12]. With the aid of learning automata, an energy efficient barrier coverage algorithm is proposed to select minimum number of required nodes to monitor barriers in deployed network [10]. An energy-efficient adaptive resource scheduler for Networked Fog Centers is proposed in [11] for real-time vehicular cloud services to meet quality-of-service (QoS) requirements. An energy-efficient stable election routing algorithm is presented in [12] to maintain balanced energy consumption of nodes in a wireless sensor network.

Despite the fruitful research on the deployment of low-power small BS, several open issues remain unsolved which are the obstacles for achieving high energy-efficient multi-tier HetNets. One of the fundamental challenges is the energy limitation of sensors. In general, most of the nodes are powered by battery with limited energy storage and lifetime. Hence, wireless powered communication networks (WPCNs), where BSs in HetNets can use energy harvest (EH) and wireless power transfer (WPT) technique, is a promising solution to prolong the lifetime of energy-limited nodes [13,14,15,16,17,18]. In [13], power allocation is designed for the maximization of energy efficiency of an energy harvesting system relying on renewable natural energy sources such as solar and wind. However, renewable energy sources are intermittent and the communication devices may not always be able to harvest sufficient energy for supporting their energy consumptions. As an alternative, wireless energy transfer technology allows low-power nodes to harvest energy from their received radio frequency (RF) signals to recharge their batteries and prolong their lifetimes [14]. In [15], three policies of wireless power transfer are proposed to guarantee secure communications in large scale cognitive cellular networks. Considering direct communication in underlying downlink cellular networks, the harvested energy in the RF is exploited to prolong the lifetime of nodes link and obtain the maximum the sum-rate by joint resource block and power allocation [16]. In [17], the authors analyze the outage probability and the corresponding optimal offloading bias in an EH HetNet which can provide energy for communication. The optimal EE of cellular communication and device-to-device (D2D) communication hybrid network is investigated by jointly the time allocation, spectrum allocation and the power control when D2D transmitters obtain energy by wireless power transfer [18].

The second key problem is high energy efficiency transmission schemes in WPCNs. Compared to the natural renewable sources available for EH technology, BS using WPT can offer a more controllable and relatively stable energy source [19,20,21,22,23]. In [19], the authors studied the switching between acting as an information relay and an energy harvesting node. In [20], the authors focus on designing appropriate transmission policies to improve the global EE in sensor networks with simultaneous wireless information and power transfer (SWIPT). An optimal maximum throughput approach for energy beamforming, receive beamforming, and time-slot allocation jointly optimization is proposed in [21]. The Ginibre model is adopted in [22] to analyze the performance of self-sustainable communications over cellular networks considering the RF energy harvesting rate and the energy outage probability. A unified framework is proposed in [23] to investigate the impact of SWIPT on the system performance with both time splitting and power splitting schemes. However, the optimal tradeoff between receiving information and harvesting energy from BSs in WPCNs has not been reported yet. With the help of WPT technology, transmitting information and harvesting energy can be added to the new fairness criterion.

Another fundamental issue of multi-tier heterogeneous WPCNs is to associate a sensor with a particular serving BS. In practical systems, the received power based user association rule is the most commonly adopted one [24], where a sensor will choose to associate with the specific BS providing the maximum received signal strength (max-RSS). This user association policy is made according to the quality of service (QoS) requirements of the devices with the goal of maximizing the capacity of communication. As far as the problem formulation is to maximize the EE of networks, the max-RSS user association decisions may not be optimal. Considering that BSs in macrocells have a significantly higher transmit power than those in small cells, the access network energy consumption is typically higher when a user is associated with a macrocell. On the other hand, resource allocation with the consideration of fairness has become an important issue in communication networks [25]. For a traditional user fairness problem, each user should be allocated with a certain amount of radio resources via a careful design of scheduling. In multi-tier heterogeneous WPCNs, the fairness problem arises not only in scheduling within a traditional cell but also in the user association decision among BSs in different tiers. Thus, the optimal energy efficiency proportional fairness of multi-tier heterogeneous WPCNs by adjusting the user association policy should also be investigated.

To address the above issues in this paper, we propose a framework for modeling and evaluating the downlink energy efficiency proportional fairness of multi-tier heterogeneous WPCNs. Our model takes into account the information transmission for active nodes and WPT for inactive nodes at BSs. Based on this system model, we propose a proportionally fair utility function to evaluate the average EE of nodes. With the aid of stochastic geometry, the average transmission rate and the harvesting energy of nodes in WPCNs are firstly analyzed to evaluate the impact on average EE. Consequently, we could characterize the fairness utility function averaged over BS locations and fading channels, so it does not depend on a specific network realization. Next, noted that the fairness utility function is non-concave for user association bias, we show that the fairness utility function can be relaxed as a concave function with respect to the receive threshold and user association bias. Then, by maximizing the relaxed utility function, we can obtain the optimal receive threshold and user association bias for the tradeoff between information transmission and power transfer. This allows us to derive an efficient iterative algorithm for obtaining the optimal solution. Exploiting alternating optimization for joint association probability and receive threshold, we also propose an efficient iterative algorithm for obtaining the suboptimal solution and reducing the compute complexity of iterative algorithm. The main contributions can be summarized as:

1. The average transmission rate and harvesting energy of nodes in WPCNs are analyzed and the impact of user association bias and receive threshold on EE of networks is revealed.
2. In the downlink multi-tier heterogeneous WPCNs, there exists an optimal receive threshold for maximizing the EE proportionally fair utility function in any tier.
3. An efficient iterative algorithm for obtaining the optimal solution of proportionally fair utility function for downlink nodes is proposed.

The rest of this paper is organized as follows. In Section 2, the system modeled with a stochastic geometry is presented. Considering the transmission rate and harvested energy, the energy efficiency proportional fairness utility function is introduced. The impact of average transmission rate and the harvested energy of nodes in WPCNs on the average EE proportional fair utility function are analyzed in Section 3. The receive threshold and user association bias optimization for the maximization of the system EE proportional fair utility function is designed in Section 4. By exploiting an alternating programming, a novel low-complexity iterative algorithm was proposed to obtain the sub-optimal solution of this problem. The derived results are validated in Section 5 by simulation results, where the impact of various system parameters on the proportionally fair utility function is illustrated. Finally, Section 6 concludes the paper.

A list of the symbols employed in this paper is given in Table 1.

## 2. System Model

### 2.1. Network Model

**Figure 1:**
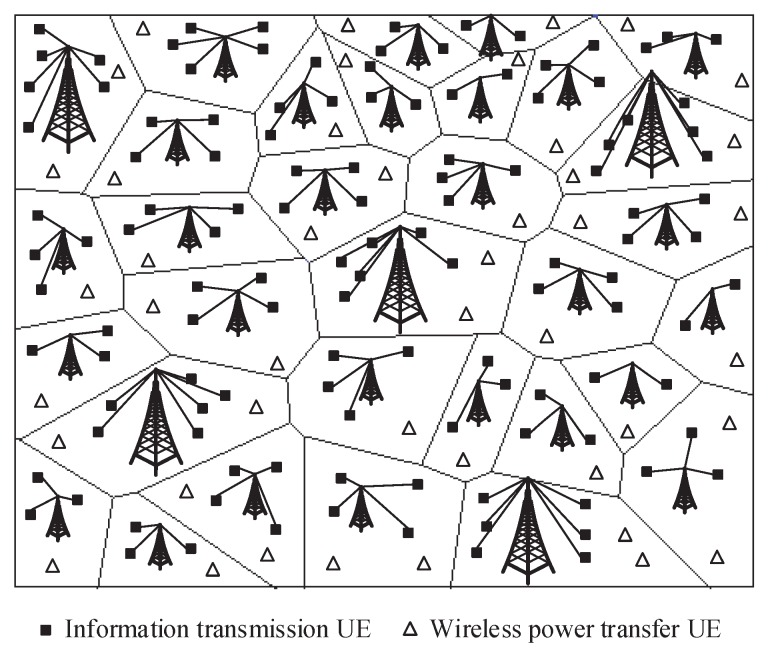
System model of a multi-tier heterogeneous wireless powered communication network (WPCN).

As shown in Figure 1, we consider a multi-tier heterogeneous WPCN in which the BSs of each tier are spatially distributed to provide seamless access service over the whole $R^{2}$ plane. Let $m_{i}^{\left(k\right)}$ denote the location of BS *i* in tier $k\in \left\{1,\ldots ,K\right\}$. The location of BSs in tier *k* is denoted by $\Phi_{k}=\left\{m_{i}^{\left(k\right)};i=1,2,3,\ldots \right\}$, where the transmit power of BSs in tier *k* is $P_{t,k}$ and the bias factor is $B_{k}$, where $B_{k}\geq 1$ for any $k\in \left\{1,\ldots ,K\right\}$. The bias factor is the factor which is used to adjust the association probability among different tiers in multi-tiers HetNets. The transmit power of BSs can multiply the bias factor to obtain “more” transmit power to let more users associate with the BSs. However, the actual transmit power of BSs cannot increase by multiplying the bias factor. The achievable rate of information transmission and the amount of wireless power transfer will not be impacted by the bias factor. We assume $\Phi_{k}$ following an independent homogeneous Poisson point process (PPP) with density $\lambda_{k},k\in \left\{1,\ldots ,K\right\}$. The superposition of *K* tiers can be denoted as $\Phi ={⋃}_{k}\Phi_{k}$ and forms a weighted Poisson Voronoi tessellation due to the inhomogeneous transmit powers of the BSs in different tiers.

In addition, the nodes obey a homogeneous PPP $\Phi_{u}$ with density $\lambda_{u}$, which is independent of $\Phi_{k}$. We assume that $\lambda_{u}$ is large enough so that each BS serves at least one associated UE per channel, i.e., $\lambda_{u}\gg \lambda_{k}$ for any $k\in \left\{1,\ldots ,K\right\}$. That is, the downlink channels are fully occupied such as in saturated conditions. Nodes in the downlink will receive the interference signal from other BSs serving their own nodes on the same channels. Each node is assumed to be equipped with single-antenna and a rechargeable battery with a large storage. In the downlink multi-tier heterogeneous WPCNs, each node can receive the desired information from its associated BS and harvest energy from both the serving BS and the interfering BSs. It can be seen from the Figure 1 that some of UEs in WPCN receive the information and others harvest energy from the BSs over the whole plane.

### 2.2. Path Loss and User Association

We consider both small- and large-scale propagation effects in the channel model. In particular, given a transmitter at $x\in R^{2}$, the receiving power at $y\in R^{2}$ is given by $P_{t,x}Ah_{x,y}L^{-1}\left(x,y\right)$, where $P_{t,x}$ is the transmit power, *A* is a propagation constant, $h_{x,y}$ denotes the fading channel power due to multi-path propagation from *x* to *y*. Moreover, $L\left(x,y\right)={∥x-y∥}^{\alpha }$ models the channel variations caused by path loss $∥x-y∥$, where α is the path loss exponent and $∥x-y∥$ denotes the Euclidean distance between *x* and *y*. We consider Rayleigh multipath fading and log-normal shadowing, i.e., $h_{x,y}$ ∼ exp$\left(1\right)$ is exponentially distributed with unit mean power. For deriving the analytical results, it is assumed that it is rare for the path loss exponent to vary across different tiers.

Each node associates to the BS that provides the maximum average bias-received-power (BRP). For example, the node located at *y* is associated to the BS at *x* in tier *y* if and only if $P_{t,k}L^{-1}\left(x,y\right)B_{k}\geq P_{t,j}L_{min,j}^{-1}\left(y\right)B_{j}$ for j=1,…,K. When $B_{k}$ is constant for any *K*-tier, the biased cell association policy will reduce to maximum average received power policy. The maximum average BRP association is stationary [26], i.e., the association pattern is invariant under translation with any displacement. According to the Palm theory [27], the analytical results of a typical cell $C_{0}$ in tier *k* can be extended to other cells $C_{i}\left(i=1,2,\ldots \right)$ in the same tier. Therefore, we only need to focus on the cell $C_{0}$ for the analysis in the remainder of this paper.

On the condition that the path loss exponent is the same in all tiers, the probability of a node associated with *k*-th tier $A_{k}$ under maximum average BRP policy can be obtained as [28]

(1) Ak=λkPt,kBk22αα∑j=1KλjPt,jBj22αα.

We can see that the association probability depends on the cell association biases, the densities of BSs, and the transmit powers in each tier.

### 2.3. Coverage Rate and Energy Harvesting

For multi-tier heterogeneous WPCNs, the thermal noise is usually negligible as compared to the interference. Hence, we consider the signal-to-interference ratio (*SIR*) instead of SINR in this work. When a randomly chosen node (termed the typical node) located at the origin *O* associates with its serving BS (termed the typical BS) at cell $C_{i}$ in tier *k*, the *SIR* of typical UE expression can be obtained based on the channel path loss model as

(2) $$\begin{array}{ccc} SIR_{k} & = & \frac{P_{t,k}h_{k,x}L_{k,x}^{-1}}{\sum_{j=1}^{K}\sum_{y\in \Phi_{j\setminus x}}P_{t,j}h_{j,y}L_{j,y}^{-1}}, \end{array}$$

where $L_{k,x}$ is the distance between the typical node and its serving BS in tier *k*, $L_{j,y}$ is the distance between the typical node and interference BS in tier *j*.

In this paper, we consider the case when a node associated with cell *j* in tier *k*. Then, the coverage probability of the node is defined as [29]

(3) $$\begin{array}{ccc} C_{k} & = & P\left(SIR_{k}>\theta_{k}\right), \end{array}$$

where $\theta_{k}$ is the *SIR* threshold of nodes associated to the tier *k* in the network. By adopting the coverage probability, the node cannot transmit information when $SIR_{k}\leq \theta_{k}$. It is expected that when nodes with a better transmit condition or less stringent QoS requirement will enable information transmission. Moreover, the *SIR* and receive threshold play important roles in the analysis of EE proportionally fairness since they can affect the coverage probability.

On each spectrum resource block, we assume that if the *SIR* is less than θ, the node does not allocate any rate and it just receives the wireless power transfer from all the BSs in the network. Otherwise, it will be served with a constant rate to obtain the fairness of information transmission. The downlink transmission rate of the node associated with tier *k* under this model is given by

(4) $$\begin{array}{ccc} R_{k} & = & \frac{W}{N}C_{k}\log_{2}\left(1+\theta \right), \end{array}$$

where *N* is the total number of active nodes sharing the downlink spectrum resource, and *W* is the total bandwidth allocated to the nodes in the WPCNs.

When the nodes cannot transmit information, they can harvest energy from the BSs in whole plane to save the energy consumption of networks, the received power of the nodes associated to tier *k* is

(5) $$\begin{array}{ccc} P_{EH,k}\; & = & \;\eta \left(1-C_{k}\right)P_{EH\_t,k}\\ & = & \;\eta \left(1-C_{k}\right)\sum_{j=1}^{K}\sum_{y\in \Phi_{j}}P_{t,j}h_{j,y}L_{j,y}^{-1}, \end{array}$$

where η is the energy harvesting efficiency of the nodes [30].

### 2.4. Energy Efficiency Proportional Fairness Utility Function

Considering that both information transmission and wireless power transfer contribute to the energy efficiency of networks, we are interested in the typical node since its average performance represents the average system performance. The energy efficiency of typical node associated with BSs in tier *k* can be defined as $\frac{R_{k}}{P_{t,k}+P_{s,k}-P_{EH,k}}$, where $P_{t,k}$ and $P_{s,k}$ are the transmit power and static power of BSs in tier *k*, respectively.

Considering that both information transmission and wireless power transfer can improve the energy efficiency, we use the proportional fairness algorithm to schedule the users for balancing the two kinds of users in WPCN. The proportional fair utility [31,32], captures the tradeoff between opportunism and user fairness, by encouraging low rate users to improve their rates while saturating the utility gain of high-rate users. According to the statement in [33], the proportional fairness should be defined as the sum of logarithm function. Therefore, the average energy efficiency-based proportional fairness of the typical node can be described as

(6) $$\begin{array}{ccc} U_{EE,k} & = & E\left[\log\left(\frac{R_{k}}{P_{t,k}+P_{s,k}-P_{EH,k}}\right)\right]. \end{array}$$

Considering the fairness of typical node among its serving BS (termed the typical BS), the average energy efficiency-based proportional fairness of the typical node in the multi-tier wireless network is

(7) $$\begin{array}{ccc} U_{EE} & = & \sum_{k=1}^{K}A_{k}U_{EE,k}, \end{array}$$

where $A_{k}$ is the probability of a node associated with BSs in *k*-th tier and is described in (1).

Note that the utility of each node is based on its average energy efficiency averaged over the fading channel. The mean system utility is the average of such utilities of all users over the network topology, which is equivalent to the mean utility of the typical user according to the Palm theory.

## 3. Performance Analysis for Downlink WPCN

### 3.1. Coverage Probability

In this subsection, we will now compute the mean coverage probability of the typical user, which is defined as $E\left[\log\left(C_{k}\right)\right]$. When the typical node associates with cell $C_{i}$ in tier *k*, the path loss between BS in cell $C_{i}$ and the typical node is $L_{k,j}=l$, the cumulative distribution function (CDF) of *SIR* of the typical node can be obtained as

(8) $$\begin{array}{ccc} E\left[C_{k}\right] & = & E_{\Phi ,h}\left[P\left(SIR_{k}>\theta_{k}\right)\right]\\ & \overset{\left(a\right)}{=} & E_{\Phi ,h}\left[P\left(\frac{P_{t,k}h_{k,i}l^{-\alpha }}{\sum_{j=1}^{K}\sum_{y\in \Phi_{j}\setminus i}P_{t,j}h_{j,y}l_{j,y}^{-\alpha }}>\theta \right)\right]\\ & = & E_{\Phi ,h}\left[P\left(h_{k,i}>\theta l^{\alpha }P_{t,k}^{-1}\left(\sum_{j=1}^{K}\sum_{y\in \Phi_{j}\setminus i}P_{t,j}h_{j,y}l_{j,y}^{-\alpha }\right)\right)\right]\\ & \overset{\left(b\right)}{=} & E_{\Phi ,h}\left[\exp\left(-\theta l^{\alpha }P_{t,k}^{-1}\left(\sum_{j=1}^{K}\sum_{y\in \Phi_{j}\setminus i}P_{t,j}h_{j,y}l_{j,y}^{-\alpha }\right)\right)\right]\\ & \overset{\left(c\right)}{=} & E_{\Phi ,h}\left[\prod_{j=1}^{K}L_{I_{j}}\left(\theta l^{\alpha }P_{t,k}^{-1}\right)\right], \end{array}$$

where $L_{I_{j}}\left(s\right)$ is the Laplace transform of $I_{j}=\sum_{y\in \Phi_{j}\setminus i}P_{t,j}h_{j,y}l_{j,y}^{-\alpha }$. (a) is derived from the expression of *SIR* in (3); (b) is due to $h_{x}$∼$\exp\left(1\right)$ is exponentially distributed with unit mean power; (c) is from the Campbell theorem for PPP.

The Laplace transform of the total interference power from the BSs in tier *j* can calculated as

(9) EΦ,h∏j=1KLIjθlαPt,k−1=EΦ,hexpθlαPt,k−1Pt,j∑y∈Φj∖ihj,ylj,y′−α=Ehexp2πλjθlαPt,k−1Pt,j∫Pt,jBjPt,kBk11ααl∞1−Lhj,yy−αydy=(d)exp2πλjθlαPt,k−1Pt,j∫Pt,jBjPt,kBk11ααl∞1−11+y−αydy≤exp2πλjθlαPt,k−1Pt,j∫Pt,jBjPt,kBk11ααl∞y−αydy=exp−2πλjθl2α−2Pt,jPt,k22ααBjBk22αα−1,

(d) is derived from associated rule that when the node is associated with the *k*-th BS tier, the length of interfering links and that of the serving link has the following relationship lj,i≥lk,0Pt,jBjPt,kBk11αα, for any *i* and *j*.

Considering $\log\left(x\right)$ is a concave function, we can obtain from the Jensen inequality and (9)

(10) ElogCk≤logECk=−2πλjθl2α−2Pt,jPt,k22ααBjBk22αα−1=(e)−2πθkl2Pkα−2λkα2+1Akα2+1∑j=1KAjα+1Pjλjα2,

(e) is derived from the relationship described in (1).

It should be noted that the mean logarithm of coverage probability of the typical user is affected by the receive threshold and user association probability. It is reasonable that if the receive threshold increases, some nodes at the edge of covered region will be inactive and harvest energy. Meanwhile, the adjustment of user association probability will impact on the coverage probability of the typical node. This is because more nodes receive information from BSs when the bias of BSs increases.

### 3.2. Average Number of Active Node

Due to the cells in multi-tier heterogeneous WPCNs form a weighted Poisson Voronoi tessellation, the probability density function (PDF) of the size of the normalized Voronoi cell is approximated by a two-parameter gamma function [34]

(11) $$\begin{array}{ccc} f_{s}\left(x\right) & = & \frac{3.5^{3.5}}{\Gamma \left(3.5\right)}x^{3.5}e^{-3.5x}. \end{array}$$

For a given cell size, the number of users associated with a BS follows a Poisson distribution with parameter $\frac{A_{k}\lambda_{u}}{\lambda_{k}}$. The probability mass function (PMF) of the number of users associated with a BS in tier *k* can be derived from (11) as

(12) PNk=n=∫0∞Akλuxλknn!e−AkλuxλkfSxdx=∫0∞Akλuxλknn!e−Akλuxλk3.53.5Γ3.5x3.5e−3.5xdx=3.53.5Γn+3.5AkλuAkλuλkλknn!Γ3.5AkλuAkλuλkλk+3.5n+3.5.

Condition on the typical node associated with a BS in tier *k*, the PMF of the number of other users associated with the BS can be obtained similarly to (12)

(13) PN′k=n=3.53.5Γn+4.5AkλuAkλuλkλknn!Γ3.5AkλuAkλuλkλk+3.5n+4.5.

The average bandwidth allocated to the typical user associated with BS in tier *k* is

(14) EWN=W·E1N′k+1=W·∑n=0∞1n+1PN′k=n=W·∑n=0∞1n+13.53.5Γn+4.5AkλuAkλuλkλknn!Γ3.5AkλuAkλuλkλk+3.5n+4.5=WλkAkλu∑n=1∞3.53.5Γn+3.5AkλuAkλuλkλknn!Γ3.5AkλuAkλuλkλk+3.5n+3.5=(f)WλkAkλu1−PNk=0=WλkAkλu1−3.53.5AkλuAkλuλkλk+3.53.5≈(g)WλkAkλu,

where (f) is obtained from the PMF of the number of users described in (12), and (g) is obtained due to the assumption $\lambda_{u}\gg \lambda_{k}$.

### 3.3. Harvested Energy

When the received *SIR* of UE is less than θ, it will harvest energy from BSs in the whole plane. The average harvest energy can be calculated as

(15) PEH_t,k=∑j=1KE∑y∈ΦjPt,jhj,yLj,y−1=(h)∑j=1K2πλj∫0∞1−Lhj,yPt,j−αy−αydy=∑j=1K2πλj∫0∞1−11+Pt,jy−αydy=∑j=1K2π2λjPt,j22αααsin2π2παα,

where (h) is from the Campbell theorem for PPP.

It should be noted that the average harvested energy is not impacted by user association probability and receive threshold, but the density of users and the transmit power of BSs. This is because the received power of nodes is affected by the distance from the BSs and the transmit power of BS. However, the bias of BS cannot affect the actual receive power of nodes.

## 4. Utility Optimization

### 4.1. Problem Formulation

For the considered system, the energy efficiency proportional fairness maximization problem can be mathematically formulated as:

(16) $$\begin{array}{cc} \max_{A_{k},\theta_{k}} & \;\;U\left(A_{k},\theta_{k}\right)\\ s.t. & \;\;C_{1}:\theta_{k}\geq 0,\forall k,\\ & \;\;C_{2}:\sum_{k=1}^{K}A_{k}=1,\\ & \;\;C_{3}:A_{k}>0,\forall k, \end{array}$$

where $C_{1}$ are non-negative constraints of receive threshold variables for any tier, $C_{2}$ ensures all active nodes associate with network, $C_{3}$ is the BSs in any tier at least serving for a active node.

This problem does not have a closed-form solution and it is not convex in general. Then, we will relax the objective function in the special case and solve the problem. Considering that the $\log\left(x\right)$ is a concave function, we can obtain

(17) ElogWWNN≤logEWWNN=logWλkAkλu.

The left side of (17) is due to Jensen inequality and the right side is from (14). Meanwhile, due to function $\log\left(\frac{C_{k}}{P_{t,k}+P_{s,k}-\eta \left(1-C_{k}\right)P_{EH,k}}\right)$ is concave function for $C_{k}$, we can obtain that

(18) $$\begin{array}{c} E\left[\log\left(\frac{C_{k}}{P_{t,k}+P_{s,k}-\eta \left(1-C_{k}\right)P_{EH,k}}\right)\right]\\ \leq \log\left[E\left(C_{k}\right)\right]-\log\left(P_{t,k}+P_{s,k}-\eta \left(1-E\left(C_{k}\right)\right)P_{EH,k}\right). \end{array}$$

Substituted (17) and (18) into (6), we can obtain that

(19) $$\begin{array}{c} U\left(A_{k},\theta_{k}\right)\leq U_{r}\left(A_{k},\theta_{k}\right)\\ =\sum_{k=1}^{K}A_{k}\log\left(\frac{W\lambda_{k}}{A_{k}\lambda_{u}}\right)+\sum_{k=1}^{K}A_{k}\log\left(\log\left(1+\theta_{k}\right)\right)+\sum_{k=1}^{K}A_{k}\log\left[E\left(C_{k}\right)\right]\\ -\sum_{k=1}^{K}A_{k}\log\left(P_{t,k}+P_{s,k}-\eta \left(1-E\left(C_{k}\right)\right)P_{EH,k}\right). \end{array}$$

This upper-bounded energy efficiency proportional fairness utility function is formulated as

(20) $$\begin{array}{cc} \max_{A_{k},\theta_{k}} & \;\;U_{r}\left(A_{k},\theta_{k}\right)\\ s.t. & \;\;C_{1}:\theta_{k}\geq 0,\forall k,\\ & \;\;C_{2}:\sum_{k=1}^{K}A_{k}=1,\\ & \;\;C_{3}:A_{k}>0,\forall k. \end{array}$$

### 4.2. Property of the Problem

The object function of Problem (20) is also non-convex, we will investigate the properties of the problem in this subsection. We can solve the considered problem according to the following Theorems.

**Theorem** **1.** For any tier in the downlink multi-tier heterogeneous WPCNs, there exists an optimal receive threshold $\theta_{k}^{*}$ for maximizing the energy efficiency proportional fairness utility function in tier k.

**Proof.**  According to (19), it can be easily proved that

(21) $$\begin{array}{c} \frac{\partial^{2}\log\left(\log\left(1+\theta_{k}\right)\right)}{\partial \theta_{k}^{2}}=-\left[\frac{1}{\log^{2}\left(1+\theta_{k}\right)}+\frac{1}{\log\left(1+\theta_{k}\right)}\right]\frac{1}{{\left(1+\theta_{k}\right)}^{2}}<0. \end{array}$$

Hence, $\log\left(\log\left(1+\theta_{k}\right)\right)$ is a concave function of $\theta_{k}$ .

Meanwhile, we can obtain that $\log\left[E\left(C_{k}\right)\right]$ is an affine function of $\theta_{k}$ from (10).

Substituting (10) and (15) into (19) yields

(22) logPt,k+Ps,k−η1−ECkPEH,k=logPt,k+Ps,k−η1−exp−2πθkl2Pkα−2λkα2+1Akα2+1∑j=1KAjα+1Pjλjα2∑j=1K2π2λjPt,j22αααsin2π2παα,

is a convex function of $\theta_{k}$. Therefore, $U_{EE,k}\left(\theta_{k}\right)$ is a concave function for $\theta_{k}$. ☐

The optimal receive threshold can be obtained via taking the first order derivative of $U_{EE}$ with respect to $\theta_{k}$ as

(23) $$\begin{array}{c} \frac{\partial U_{EE}\left(\theta_{k}\right)}{\partial \theta_{k}}=0 \end{array}$$

Hence, we could get the optimal receive threshold by the expression as follows

(24) $$\begin{array}{c} \bar{P}+\beta \exp\left(-h\left(A_{k}\right)\theta_{k}\right)=\bar{P}h\left(A_{k}\right)\left(1+\theta_{k}\right)\log\left(1+\theta_{k}\right), \end{array}$$

where $\bar{P}=P_{t,k}+P_{s,k}-\eta \sum_{j=1}^{K}\frac{2\pi^{2}\lambda_{j}p_{j}^{\frac{2}{\alpha }}}{\alpha \sin\left(\frac{2\pi }{\alpha }\right)}$ which is independent of $A_{k}$, $\beta =\eta \sum_{j=1}^{K}\frac{2\pi^{2}\lambda_{j}p_{j}^{\frac{2}{\alpha }}}{\alpha \sin\left(\frac{2\pi }{\alpha }\right)}$, and $h(A_{k})$ can be obtained in the Appendix A.

Theorem 1 shows that if in any tier where the user association probability of nodes $A_{k}$ is fixed, we can find an optimal receive threshold $\theta_{k}^{*}$ to obtain the tradeoff of nodes between receiving information and harvesting energy. Note that the receive threshold includes two special cases: (i) when $\theta_{k}=0$, the receive threshold is so low that all nodes can receive information regardless of the QoS requirement from information transmission; (ii) when $\theta_{k}\to \infty$, the receive threshold is so high that all nodes should harvest energy regardless of the distance between nodes and BSs. From (13), we can obtain the optimal receive threshold $\theta_{k}^{*}>0$. Therefore, the optimal receive threshold $\theta_{k}^{*}$ brings to the balance for node to receive information and harvest energy.

**Figure 2:**
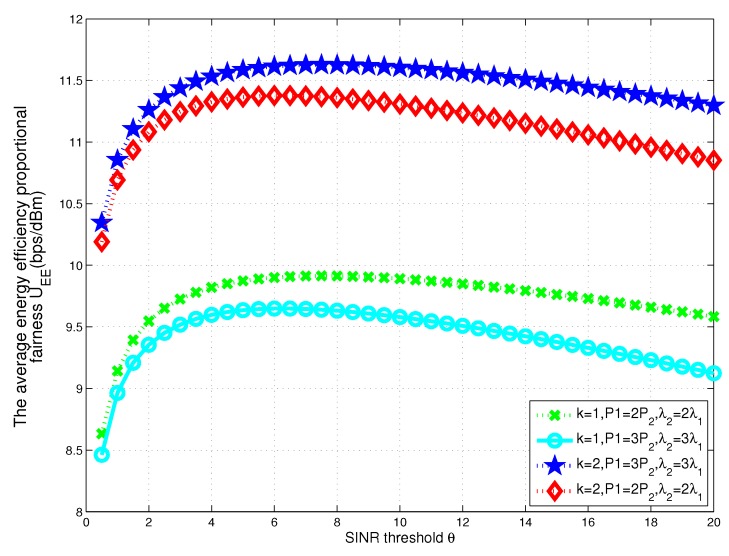
The relationship between energy efficiency proportional fairness utility function and the receive threshold for different transmit powers and densities of BSs in two-tier heterogeneous WPCNs.

To reveal the relationship between energy efficiency proportional fairness utility function and the receive threshold in a two-tier heterogeneous WPCNs, we show some numerical results for different transmit powers and densities of BSs in two-tier heterogeneous WPCNs in Figure 2. Numerical and analytical results show that there exists an optimal threshold $\theta^{*}$ for maximizing the utility function in each tier.

We can obtain the relationship between optimal receive threshold $\theta_{k}^{*}$ and the user association probability $A_{k}$ as the following lemma.

**Lemma** **1.** The optimal receive threshold $\theta_{k}^{*}$ in any tier decreases with the user association probability $A_{k}$.

**Proof.**  This lemma can be proved by exploiting the implicit function theorem which can be found in Appendix A. ☐

It should be noted that when the user association probability $A_{k}$ increases, more nodes will be associated with the BSs. In this case, the average transmit rate will decrease due to the bandwidth allocated to each node reducing. Therefore, the decreasing of optimal receive threshold will increase the energy efficiency proportional fairness utility function.

**Theorem** **2.** The relaxed energy efficiency proportional fairness utility function is concave for association probability in any tier.

**Proof.**  This theorem can be proved by exploiting the implicit function theorem which can be found in Appendix B. ☐

### 4.3. Sub-Optimal Algorithm Design

According to the above Theorem, we can obtain the solution by combining the solutions of the two sub-problems. A two-step sub-optimal algorithm is proposed: (1) Initialize the receive threshold of all nodes is zero, which means all nodes receive information. Adjusting the bias of BSs in each tier to obtain the optimal energy efficiency proportional fairness; (2) Fix the optimal bias of BSs in each tier, adjusting the receive threshold of nodes to obtain the optimal energy efficiency proportional fairness. Then repeat to run step 1 and 2 until the result converge reaches the optimal solution. Due to Theorem 2, the first step is solvable. Meanwhile, the second step is solvable according to Theorem 1. The detailed description of this two-step algorithm is presented in Algorithm 1.

**Algorithm 1. Suboptimal Iterative Resource Allocation Algorithm**1: Initialize the maximum number of iterations $L_{\max}$, the maximum tolerance is 0≤ε≤12: Set the associated probability $A_{k}=A_{k}^{\left(0\right)}=0dB,\theta_{k}=\theta_{k}^{\left(0\right)}=0,\forall k=\left\{1,2,\ldots ,K\right\}$, and iteration index *n* = 03: **repeat** {Loop}4:  Solve the convex problem in (19) for a given set of $A_{k}^{\left(n\right)}$ and obtain the optimal receive threshold $\left\{A_{k}^{\left(n\right)},\theta_{k}^{\left(n+1\right)}\right\}$5:  Solve the convex problem in (19) for a given set of $\theta_{k}^{\left(n\right)}$ and obtain the optimal association probability $\left\{A_{k}^{\left(n+1\right)},\theta_{k}^{\left(n\right)}\right\}$6:  **if**
$\left|A_{k}^{n+1}-A_{k}^{n}\right|<\varepsilon$ and $\left|\theta_{k}^{n+1}-\theta_{k}^{n}\right|<\varepsilon$
**then**7:   Convergence=true8:   **return**
$\left\{A_{k}^{*},\theta_{k}^{*}\right\}=\left\{A_{k}^{\left(n+1\right)},\theta_{k}^{\left(n+1\right)}\right\}$ and $U^{*}\left(A_{k},\theta_{k}\right)=U\left(A_{k}^{*},\theta_{k}^{*}\right)$9:  **else**10:   n=n+111:   Convergence = **false**12:  **end if**13: **until** Convergence = **true** or $n=L_{\max}$

The problem (20) can also be solved to obtain the optimal receive threshold and user association probability by performing optimal brute-force two-dimensional search. However, the computational complexity of the proposed optimal two-dimensional search scheme is upper bound by $O\left(\frac{\theta_{max}}{M}\cdot \frac{K^{2}}{N}\right)$, where $\theta_{max}$ is the maximal value of the receive threshold, *M* is the step size of the one-dimensional search on the receive threshold, *K* is the number of tiers in the network, and *N* is the step size of the one-dimensional search on user association probability. It can be seen that the complexity of the algorithm is relatively higher due to the two-dimensional search performed by the loop, and the complexity increases nonlinearly as the search step decreases.

It is clear from Algorithm 1 that in the first step of the proposed sub-optimal algorithm, there are K iterations. In each iteration, the number of comparisons required to find the best association probability of that tier is $\log_{2}N$ by adopting the binary search. In the second step, for the fixed association probability, the optimal receive threshold can be obtained by solving the Formula (24). Therefore, the maximum number of comparisons required by our proposed sub-optimal algorithm is $O\left(K\log_{2}N+K-1\right)$. Compared with the optimal brute-force search, it can be clearly seen that by relaxing the objective function and decoupling the problem into two sub-problems, the computational complexity is significantly reduced. In addition, the suboptimal algorithm obtains the suboptimal solution of the corresponding optimization problem, and the performance gap between the suboptimal and optimal will be shown in the simulation results.

## 5. Numerical Simulations

In this section, we investigate the performance of the proposed user association and threshold scheme through simulations. In the system performance simulations, we evaluate numerically the energy efficiency proportional fairness of a two-tier downlink heterogeneous WPCNs. Unless otherwise specified, the system parameters are assumed as: path loss exponent α=4, system bandwidth *B* = 10 MHz, static power consumption of BS in each tier is assumed to be equal to $P_{s}$ = 0 dBm, the transmit power of BSs in tier 1 and 2 are $P_{t,1}$ = 41 dBm, $P_{t,2}$ = 33 dBm , the density of BSs in tier 1 and 2 is $\lambda_{1}=4\times 10^{-5}$/m${}^{2}$, $\lambda_{2}=28\times 10^{-5}$/m${}^{2}$, the bias factor of tier 1 is $B_{1}$ = 0 dB, the density of UEs is $\lambda_{u}=20\lambda_{1}$, the ratio of energy conversion for EH η=0.7.

### 5.1. Effect of the Transmit Power of BSs

**Figure 3:**
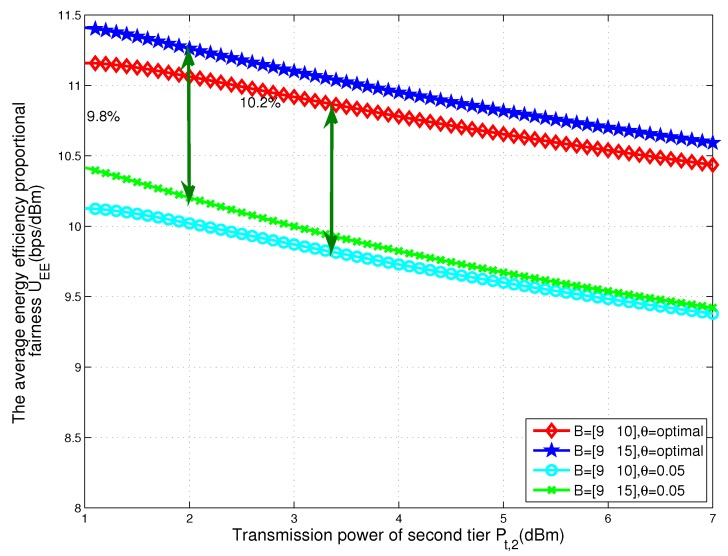
Energy efficiency proportional fairness of total two-tier heterogeneous WPCNs with respect to the transmit power of BSs in tier 2 for different values of the tier-2 cell association bias B2.

In Figure 3, we plot the energy efficiency proportional fairness of total two-tier heterogeneous WPCNs with respect to the transmit power of BSs in tier 2 for different values of the tier-2 cell association bias $B_{2}$. We can see that as the transmit power of BSs in tier 2 changes from 1 dBm to 7 dBm, the maximal energy efficiency proportional fairness decreases. This is because, as the transmit power of tier 2 increases, the energy efficiency proportional fairness of tier 2 decreases according to (7). Therefore, the total energy efficiency proportional fairness of network decreases with the increasing transmit power of BSs in tier 2. Meanwhile, due to the transmit power of BSs in tier 2 being less than that of BSs in tier 1, the energy efficiency proportional fairness of the network increases as the bias of tier 2 increases.

For comparison, Figure 3 also shows the energy efficiency proportional fairness of another fixed threshold scheme. For this baseline scheme, we adopt a fixed threshold and small enough θ = 0.05. Therefore, most of the nodes are in the heterogeneous WPCNs transmit information and the harvest energy from the wireless power transfer is small. As can be observed, the energy efficiency proportional fairness of the system with a fixed threshold is substantially lower than those of the proposed optimal scheme. It can be seen that from the energy efficiency of the network, all of the nodes associating with network is not the best strategy since the power consumption of BSs is large. We can choose optimal threshold to let some nodes harvest energy temporarily.

In the current HetNet, a lot of low-power BSs is deployed around high-power BSs. The deployment of the network can be described as the scenario that the density of BSs in tier 2 is larger than that of BSs in tier 1 and the transmit power of BSs in tier 2 is less than that of BSs in tier 1. In this case, we can obtain from the Figure 3 that for improving the energy efficiency proportional fairness of total network, when the transmit power of BSs in tier 2 is high, the bias factor of BSs in tier 2 should be large. This is because if the low-power BSs can serve more nodes, the high-power BSs should offload some traffic to low-power BSs to enhance the energy efficiency proportional fairness of total network and balance the traffic in different tiers.

**Figure 4:**
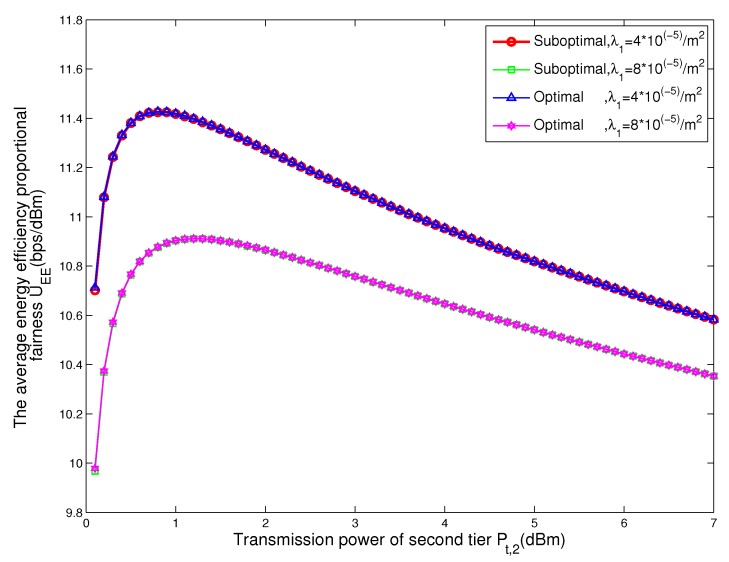
The mean energy efficiency proportional fairness with respect to the association probability of tier 2 obtained from the proposed suboptimal scheme and optimal two-dimension search scheme for different values of the transmit power of base stations (BSs).

For different values of the transmit power of BSs, Figure 4 contains the mean energy efficiency proportional fairness with respect to the density of BSs in tier 1 obtained from the proposed suboptimal scheme and optimal brute-force two-dimension search scheme. We can see that as the transmit power of BSs in tier 2 changes from 0 dBm to 7 dBm, the maximal energy efficiency proportional fairness firstly increases and then decreases. This is because if the transmit power of tier 2 is small, it will serve little nodes and a lot of nodes associated to the BSs in tier 1. Due to the BSs in tier 1 having larger transmit power than BSs in tier 2, the energy efficiency proportional fairness of the network is small. Therefore, the total energy efficiency proportional fairness of network firstly increases with the increase of the transmit power of BSs in tier 2. Meanwhile, when the transmit power of BSs in tier 2 is large enough, nodes associated with BSs in tier 2 cannot obtain higher energy efficiency. In this case, the energy efficiency proportional fairness of the network decreases as the transmit power of BSs in tier 2 increases.

As the transmit power of BSs in tier 2 changes from 0 dBm to 7 dBm, the energy efficiency proportional fairness of nodes become a bell-shaped curve when the transmit power of BSs in tier 2 increases. The maximal energy-efficiency proportional fairness increases firstly when the transmit power of BSs in tier 2 is low. This is because when the transmit power of BSs in tier 2 is low, more nodes associate with the BSs in tier 1 than in tier 2. Hence, the fairness among different tiers will be small and the energy-efficiency proportional fairness of nodes will firstly increase with the transmit power of BSs in tier 2. When the transmit power of BSs in tier 2 is high enough, more nodes associate with the BSs in tier 2 than tier 1. In this case, the fairness among different tiers will also be small. The energy-efficiency proportional fairness of nodes decrease with the transmit power of BSs in tier 2.

For comparison, Figure 4 also shows that when the density of BSs in tier 1 is $4\times 10^{-5}$/m${}^{2}$, the energy efficiency proportional fairness of network is less than that of the density of BSs in tier 1 with $8\times 10^{-5}$/m${}^{2}$. This is because nodes will associate with BSs in tier 1 when the density of BSs in tier 1 is high. However, the energy efficiency proportional fairness of tier 1 is less than that of tier 2 due to the transmit power of BSs in tier 1 being higher. Hence, the energy efficiency proportional fairness of the network decreases. Meanwhile, it can be seen that the gap of energy efficiency proportional fairness of the network between the proposed suboptimal scheme and the optimal brute-force two dimension search can be ignored.

### 5.2. Effect of the Density of BSs

**Figure 5:**
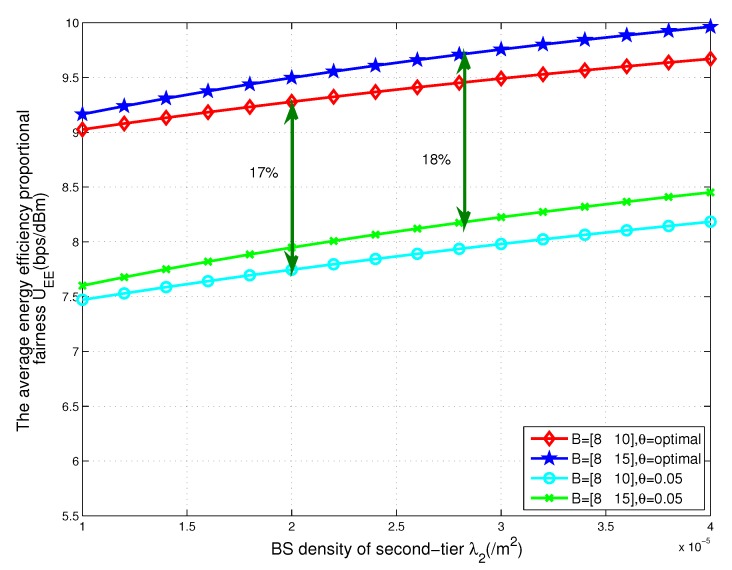
The energy efficiency proportional fairness of total two-tier heterogeneous WPCNs with respect to the density of BSs in tier 2 for different values of the tier-2 cell association bias B2.

For comparison, Figure 5 also shows the energy efficiency proportional fairness of the networks, in which the threshold is fixed as θ = 0.05. As can be observed, the energy efficiency proportional fairness of the network with fixed threshold is substantially lower than those of the proposed optimal scheme. When the threshold is small, all of the nodes associated with the network can transmit information and consume the energy of the network. This scheme will not be beneficial to the energy efficiency of the networks. We can allocate some nodes to harvest energy by improving the threshold. The nodes can recharge their batteries by harvesting the RF energy via the WPT and exploiting the harvested energy for uplink transmission.

In the current deployment of HetNet, we can obtain from Figure 5 that for improving the energy efficiency proportional fairness of the total network, when the density of BSs in tier 2 is high the bias factor of BSs in tier 2 should be small. This is because, if the number of low-power BSs increases, a lot of traffic will offload from the high-power BSs to low-power BSs. If we want to enhance the energy efficiency proportional fairness of the total network and balance the traffic in different tiers, the bias factor of BSs in tier 2 should be small.

### 5.3. Effect of the Association Probability

**Figure 6:**
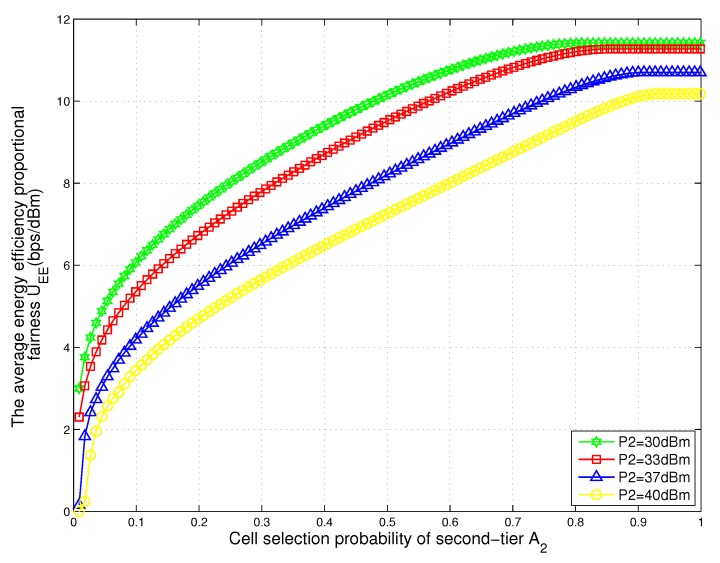
The energy efficiency proportional fairness of total two-tier heterogeneous WPCNs with respect to the association probability of tier 2 for different values of the transmit power of BSs.

In Figure 6, we plot the energy efficiency proportional fairness of total two-tier heterogeneous WPCNs with respect to the user association probability of tier 2 for different values of the transmit power of BSs. We obtain that with the increase of user association probability of tier 2, the average energy efficiency proportional fairness increases quickly and then remains unchanged. Due to the transmit power of BSs in tier 2 being less than that of BSs in tier 1, the energy efficiency proportional fairness utility function of tier 2 is larger than that of tier 1 from (7). Therefore, nodes associated with BSs in tier 2 will obtain a higher average energy efficiency proportional fairness. Meanwhile, the energy efficiency proportional fairness remains unchanged as the user association probability gets closer to 1 as shown in Figure 6. This is because when the association probability gets close to 1, the load of tier 2 is balanced such that the nodes connected with BSs in tier 1 will not transfer to BSs in tier 2. In this case, the energy efficiency proportional fairness is a constant. Furthermore, we note that the mean energy efficiency proportional fairness decreases with the transmit power of BSs in tier 2 increases for the same association probability. It can be explained from (7) that the energy efficiency proportional fairness increases with the reduction of transmit power. When the transmit power of BSs in tier 2 decreases, the energy efficiency proportional fairness in tier 2 increases and that of the total network also increases.

### 5.4. Comparison of Computation Complexity

**Figure 7:**
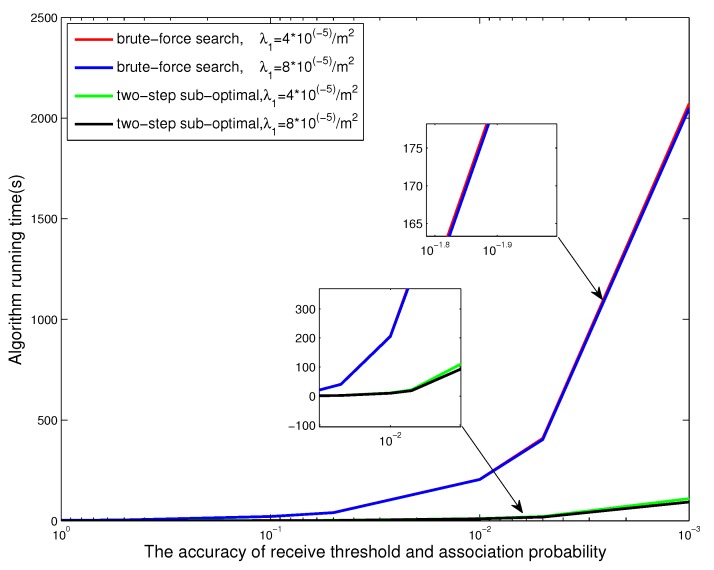
The time cost of brute-force two-dimensional search algorithm and two-step sub-optimal algorithm with respect to the accuracy of system parameters.

In order to compare the computation complexity of the two-step sub-optimal algorithm and the brute-force two-dimensional search algorithm, we show in Figure 7 the different time costs of two algorithms which obtain the maximal average energy efficiency-based proportional fairness of nodes with different accuracy of the receive threshold and association probability. Our results show that, with the increase of accuracy, the time cost on the brute-force two-dimensional search algorithm increases with increasing exponent rate. The time cost on the two-step sub-optimal algorithm increases slowly and is always less than that of the brute-force search algorithm. This simulation result shows the advantage of our proposed sub-optimal algorithm on time complexity. Meanwhile, Figure 7 further presents that the time cost of two algorithms are almost unchanged with different system parameters such as density of BSs and nodes. This is because that the time complexity of two search algorithms depends on the accuracy of the receive threshold and association probability.

## 6. Conclusions

In this paper, we studied the energy efficiency proportional fairness for downlink multi-tier heterogeneous WPCNs. The coverage probability, average number of active nodes, and the amount of energy harvesting from WPT in networks is obtained as a function of the user association probability and receive threshold with the aid of stochastic geometry. The original energy efficiency proportional fairness utility function is designed with the user association probability and receive threshold as a non-convex optimization problem. We relax the objective problem as a convex optimization problem according to the properties of receive threshold and association probability. By exploiting an alternating optimization, a novel suboptimal low-complexity iterative algorithm was proposed to obtain a suboptimal solution. Simulation results showed that the proposed optimal scheme achieves a significant improvement in system energy efficiency proportional fairness compared to the baseline scheme.

## Figures and Tables

**Table 1:** Notations used in the paper.

$\Phi_{k}$, $\lambda_{k}$	PPP of *k*-th tier BS and the corresponding density
$\Phi_{k}$, $\lambda_{u}$	PPP of UEs and the corresponding density
$P_{t,k}$, $P_{s,k}$	Dynamical transmit power and static power of *k*-th tier BS
$B_{k}$	Bias factor of *k*-th tier BS
α	Path loss exponent
$h_{x,y}$	Small scale fading between nodes located *x* and *y*
*W*, *N*	Total bandwidth and number of UEs sharing the spectrum

## Appendix A

**Proof of Lemma** **1.** From Theorem 1, we know that the first order derivative of $U_{EE}$ with respect to $\theta_{k}$ is equal to zero when $A_{k}$ is fixed. Similarly, we first write the energy efficiency of UE in $\left(7\right)$ function with respect to $\theta_{k}$ as follows

(A1) $$\begin{array}{c} U_{EE}\left(\theta_{k}\right)=\sum_{k=1}^{K}A_{k}\left\{\log\frac{W}{A_{k}N}+\log\left(\log\left(1+\theta_{k}\right)\right)+\log\left[E\left(C_{k}\right)\right]-\log\left(\bar{P}+\beta E\left[C_{k}\right]\right)\right\}, \end{array}$$

where $\bar{P}=P_{t,k}+P_{s,k}-\eta \sum_{j=1}^{K}\frac{2\pi^{2}\lambda_{j}p_{j}^{\frac{2}{\alpha }}}{\alpha \sin\left(\frac{2\pi }{\alpha }\right)}$ which is independent of $A_{k}$, $\beta =\eta \sum_{j=1}^{K}\frac{2\pi^{2}\lambda_{j}p_{j}^{\frac{2}{\alpha }}}{\alpha \sin\left(\frac{2\pi }{\alpha }\right)}$, and $C_{k}=\exp\left[-\frac{2\pi l^{2}}{\alpha -2}\sum_{j=1}^{K}\lambda_{j}{\left({\hat{p}}_{jk}\right)}^{\frac{2}{\alpha }}{\left({\hat{B}}_{jk}\right)}^{\frac{2}{\alpha }-1}\right]$. For the sake of simplicity, we make $h\left(A_{k}\right)$ a function of $A_{k}$ as shown below

(A2) $$\begin{array}{c} h\left(A_{k}\right)=\frac{2\pi l^{2}}{\alpha -2}\sum_{j=1}^{K}\lambda_{j}{\left({\hat{p}}_{jk}\right)}^{\frac{2}{\alpha }}{\left({\hat{B}}_{jk}\right)}^{\frac{2}{\alpha }-1},h\left(A_{k}\right)>0. \end{array}$$

Then we can have that $C_{k}=\exp\left[-h\left(A_{k}\right)\theta_{k}\right]$ and $\log\left[E\left(C_{k}\right)\right]=-h\left(A_{k}\right)\theta_{k}$. Moreover, we can also get the further simplified expression of $U_{EE}\left(\theta_{k}\right)$ as follows

(A3) $$\begin{array}{c} U_{EE}\left(\theta_{k}\right)\\ =\sum_{k=1}^{K}A_{k}\{\log\frac{W}{A_{k}N}+\log\left(\log\left(1+\theta_{k}\right)\right)-h\left(A_{k}\right)\theta_{k}-\log[\bar{P}+\beta \exp\left(-h\left(A_{k}\right)\theta_{k}\right)]\}. \end{array}$$

Hence, we define the function with respect to $\theta_{k}$ given below

(A4) $$\begin{array}{c} U_{EE,k}\left(\theta_{k}\right)=A_{k}\{\log\frac{W}{A_{k}N}+\log\left(\log\left(1+\theta_{k}\right)\right)-h\left(A_{k}\right)\theta_{k}-\log\left[\bar{P}+\beta \exp\left(-h\left(A_{k}\right)\theta_{k}\right)\right]. \end{array}$$

The target implicit function can be obtained via taking the first order derivative with respect to $\theta_{k}$ as

(A5) $$\begin{array}{c} \bar{P}+\beta \exp\left(-h\left(A_{k}\right)\theta_{k}\right)=\bar{P}h\left(A_{k}\right)\left(1+\theta_{k}\right)\log\left(1+\theta_{k}\right), \end{array}$$

where $\theta_{k}$ is the function of $A_{k}$ . According to the implicit function theorem, we can get the following expression

(A6) $$\begin{array}{c} \frac{d\theta_{k}}{dA_{k}}=-\frac{L_{A_{k}}}{L_{\theta_{k}}}\\ =-\frac{h{\left(A_{k}\right)}^{{}^{\prime }}\left[\beta h\left(A_{k}\right)\exp\left(-h\left(A_{k}\right)\theta_{k}\right)\theta_{k}+\bar{P}\log\left(1+\theta_{k}\right)+\bar{P}\theta_{k}\log\left(1+\theta_{k}\right)\right]}{h\left(A_{k}\right)\left[\beta \exp\left(-h\left(A_{k}\right)\theta_{k}\right)+\bar{P}\log\left(1+\theta_{k}\right)+\bar{P}\right]}\\ <0. \end{array}$$

Hence, $\theta_{k}$ decreases with $A_{k}$. ☐

## Appendix B

**Proof of Theorem** **2.** In order to evaluate the convexity of $U_{EE}$ with respect to $A_{k}$,we need to take derivative of $A_{k}$ when $\theta_{k}$ is fixed. To do so, we first write the energy efficiency of UE in $\left(7\right)$ function with respect to $A_{k}$ given below

(A7) $$\begin{array}{c} U_{EE}\\ =\sum_{k=1}^{K}A_{k}\log\frac{W}{A_{k}N}+\sum_{k=1}^{K}\{A_{k}\log\left(\log\left(1+\theta_{k}\right)\right)+A_{k}E\left[\log\left(C_{k}\right)\right]-A_{k}\log\left(\bar{P}+\beta E\left[C_{k}\right]\right)\}, \end{array}$$

where $\bar{P}$ and β are the same as proof of lemma 1. By taking a second order derivative of $A_{k}\log\frac{W}{A_{k}N}$ with respect to $A_{k}$, we get

(A8) $$\begin{array}{c} \frac{\partial^{2}\left(A_{k}\log\frac{W}{A_{k}N}\right)}{\partial A_{k}^{2}}=-\frac{1}{A_{k}}<0. \end{array}$$

Notice that the first part of the formulas is concave, we only need to prove that the latter part is the concave function. Hence, we define the remaining part as the function given below

(A9) $$\begin{array}{c} U_{EE,k}=A_{k}\log\left(\log\left(1+\theta_{k}\right)\right)+A_{k}E\left[\log\left(C_{k}\right)\right]-A_{k}\log\left(\bar{P}+\beta E\left[C_{k}\right]\right). \end{array}$$

We can obtain from (10) that $C_{k}$ is a function for $A_{k}$. Similarly, by taking a second derivative of $U_{EE,k}$ with respect to $A_{k}$, we have

(A10) $$\begin{array}{c} \frac{\partial^{2}U_{EE,k}}{\partial A_{k}^{2}}\\ =-2mN^{{}^{\prime }}\left(A_{k}\right)-A_{K}mN^{{}^{\prime \prime }}\left(A_{k}\right)+\frac{\beta m\exp\left(-mN\left(A_{k}\right)\right)N^{{}^{\prime }}\left(A_{k}\right)}{\bar{P}+\beta \exp\left(-mN\left(A_{k}\right)\right)}-{\left\{\frac{A_{k}\beta E^{{}^{\prime }}\left[C_{k}\right]}{\bar{P}+\beta E\left[C_{k}\right]}\right\}}^{{}^{\prime }}, \end{array}$$

where $m=\frac{2\pi l^{2}P_{k}\lambda_{k}^{\frac{\alpha }{2}+1}}{\alpha -2}\theta_{k}$, $N\left(A_{k}\right)=\frac{\sum_{j=1}^{K}\frac{A_{j}^{\alpha +1}}{P_{j}\lambda_{j}^{\frac{\alpha }{2}}}}{A_{k}^{\frac{\alpha }{2}+1}}$ which is from the expression of $E\left[\log\left(C_{k}\right)\right]$ as (10). Therefore, we have $E\left[\log\left(C_{k}\right)\right]=-mN\left(A_{k}\right)$, $E^{{}^{\prime }}\left[\log\left(C_{k}\right)\right]=-mN^{{}^{\prime }}\left(A_{k}\right)$ and $E^{{}^{\prime }}\left[C_{k}\right]=-m\exp\left(-mN\left(A_{k}\right)\right)N^{{}^{\prime }}\left(A_{k}\right)$. By taking the derivative of $A_{k}$,we have

(A11) $$\begin{array}{ccc} \frac{dN(A_{k})}{dA_{k}} & = & \frac{\alpha +1}{P_{k}\lambda_{k}^{\frac{\alpha }{2}}}A_{k}^{\frac{\alpha }{2}-1}-\frac{\left(\frac{\alpha }{2}+1\right)}{A_{k}^{\frac{\alpha }{2}+2}}\sum_{j=1}^{K}\frac{A_{j}^{\alpha +1}}{P_{j}\lambda_{j}^{\frac{\alpha }{2}}}, \end{array}$$

(A12) $$\begin{array}{ccc} \frac{d^{2}N\left(A_{k}\right)}{d{A_{k}}^{2}} & = & -\frac{2(\alpha +1)}{P_{k}\lambda_{k}^{\frac{\alpha }{2}}}A_{k}^{\frac{\alpha }{2}-2}+\frac{\left(\alpha +2)(\alpha +4\right)}{4A_{k}^{\frac{\alpha }{2}+3}}\sum_{j=1}^{K}\frac{A_{j}^{\alpha +1}}{P_{j}\lambda_{j}^{\frac{\alpha }{2}}}. \end{array}$$

Hence, we can get the further simplified expression of $U_{EE,k}$ as follows

(A13) $$\begin{array}{ccc} \frac{\partial^{2}U_{EE,k}}{\partial A_{k}^{2}} & = & \frac{(\alpha +2)m}{4A_{k}^{\frac{\alpha }{2}+2}}\sum_{j=1}^{K}\frac{A_{j}^{\alpha +1}}{P_{j}\lambda_{j}^{\frac{\alpha }{2}}}\left(-\frac{\alpha \bar{P}\exp\left(mN\left(A_{k}\right)\right)}{\bar{P}\exp\left(mN\left(A_{k}\right)\right)+\beta }\right)<0. \end{array}$$

Therefore, it is obvious that $U_{EE,k}$ is concave function, so $U_{EE}$ is also concave function. ☐
